# Authors’ response: SARS-CoV-2 detection by real-time RT-PCR

**DOI:** 10.2807/1560-7917.ES.2020.25.21.2001035

**Published:** 2020-05-28

**Authors:** Victor M Corman, Christian Drosten

**Affiliations:** 1 Charité – Universitätsmedizin Berlin Institute of Virology, Berlin, Germany and German Centre for Infection Research (DZIF), Berlin, Germany

**Keywords:** SARS-CoV-2, COVID-19, molecular diagnostic, real-time RT-PCR

**To the editor:** We thank Pillonel and colleagues for their comments and suggestions [1]. Their letter contains a most relevant statement: ‘These observations based on in silico alignments should be confirmed by wet-laboratory experiments, [...]’. As outlined in our initial work, oligonucleotide design at the time was based on available sequences from severe acute respiratory syndrome coronavirus (SARS-CoV) and bat-derived SARS-related CoV sequences [2]. Our strategy during establishment was to use a synthetic target for the SARS-CoV-2 E gene assay, while validating amplification of a full virus genome RNA using the RdRp assay that is specific for both, SARS-CoV and SARS-CoV-2, with the latter not being available to us in the form of an isolate or clinical sample at the time. Based on experimental validation, it later turned out that the mismatched base pairs do not reduce RT-PCR sensitivity and are not to be seen as the reason for somewhat higher Ct values with the RdRp assay as compared to the E gene assay [3]. This is rather due to the general oligonucleotide design, such as the predicted lower melting temperature of the reverse primer compared to the other oligonucleotides.

In general, mutations or unknown variations within the primer binding regions may influence the performance of RT-PCR assays, as also described for SARS-CoV-2 [4]. Oligonucleotide binding regions should be monitored continuously for their matching to circulating virus strains [5]. Providers of RT-PCR assays should announce oligonucleotide binding sites to enable this type of monitoring.
